# Infective popliteal artery aneurysm by *Streptococcus equi*: An unusual pathogen

**DOI:** 10.1016/j.jvscit.2023.101171

**Published:** 2023-03-31

**Authors:** Angelos Karelis, Torbjörn Fransson, Mac Schlyter, Talha Butt, Nuno Dias

**Affiliations:** a Vascular Center, Department of Thoracic and Vascular Diseases, Skåne University Hospital, Malmö, Sweden; b Department of Clinical Sciences Malmö, Lund University, Malmö, Sweden

**Keywords:** Atypical infection, Infective popliteal artery aneurysm, Mycotic aneurysm, Peripheral aneurysm, Popliteal aneurysm, Surgical treatment

## Abstract

We report the case of a 63-year-old man who presented with a 2-week complaint of lower extremity pain, swelling, and low-grade fever after an episode of septic arthritis in the ipsilateral knee. The investigation showed a rapidly expanding popliteal artery aneurysm (PAA). The rare clinical entity of an infective PAA was suspected and was confirmed by the cultures obtained at the right femoropopliteal bypass with an autologous vein graft and subtotal resection of the aneurysm sac. *Streptococcus equi* was identified as the primary pathogen, which, to the best of our knowledge, has not been previously described for an infective PAA.

Infective aneurysms have been described in virtually every arterial bed and can occur as primary or secondary infections, with a predisposition to a location in the abdominal aorta.^1^ Infective aneurysms can be due to bacteremia, local injury with inoculation, spread from neighboring tissues,^2^ or septic emboli, usually secondary to endocarditis.^3^ To date, <50 cases of primary infective PAA have been reported in the literature, with none due to *Streptococcus equi,* a species of gram-positive, coccoid bacteria isolated from the upper respiratory tract of horses. The patient provided written informed consent for the report of his case details and imaging studies.

## Case report

**Fig 1:**
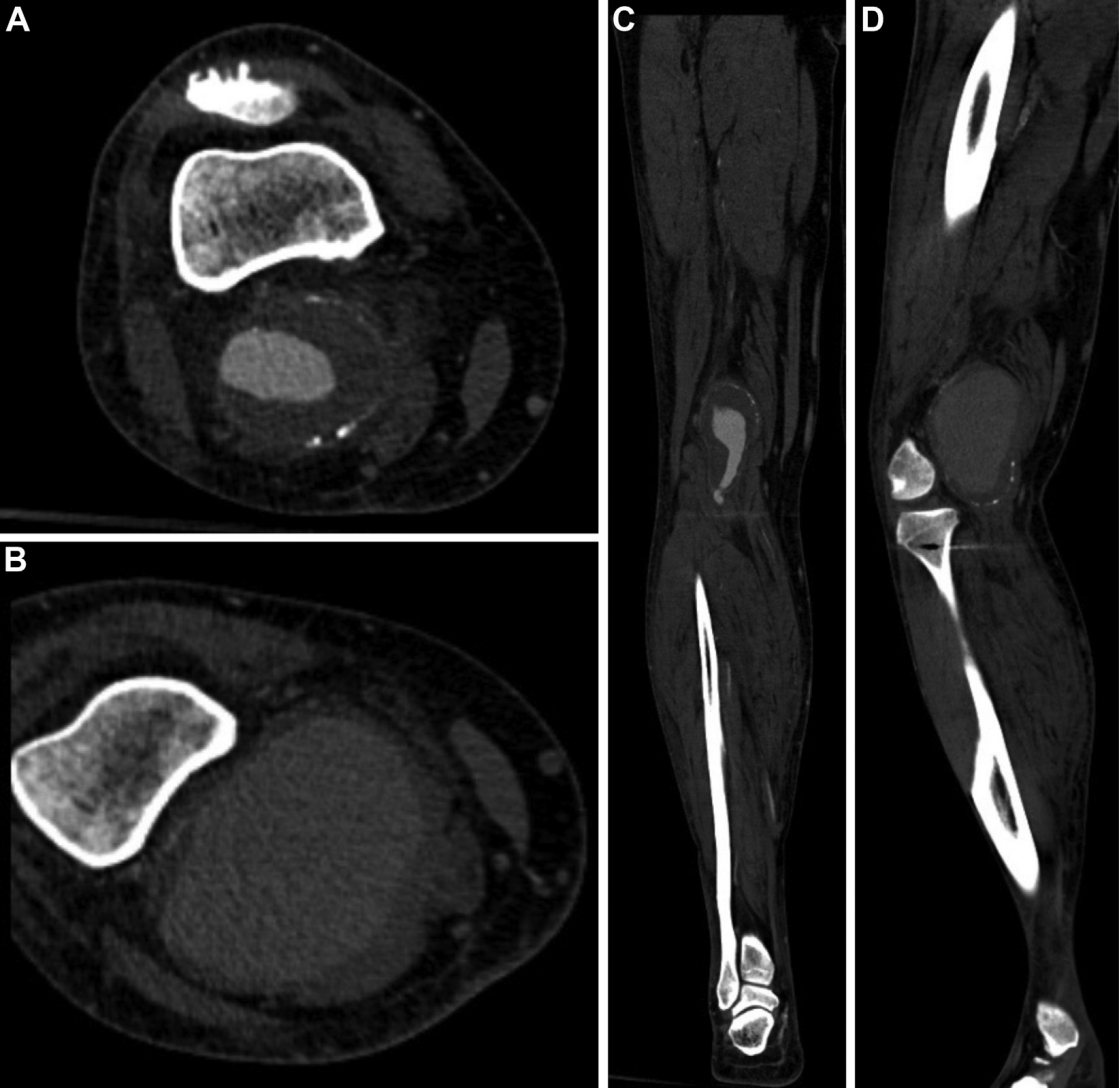
Computed tomography angiographic images of the right popliteal artery aneurysm (PAA) on axial **(A** and **D)** and coronal **(B** and **C)** projections demonstrating rapid expansion from 46 mm **(A** and **B)** to 81 mm **(C** and **D)** within a 2-week period. The aneurysm diameter was measured on the axial images perpendicular to the flow line to the largest diameter of the vessel with three-dimensional reconstructed computed tomography images.

**Fig 2:**
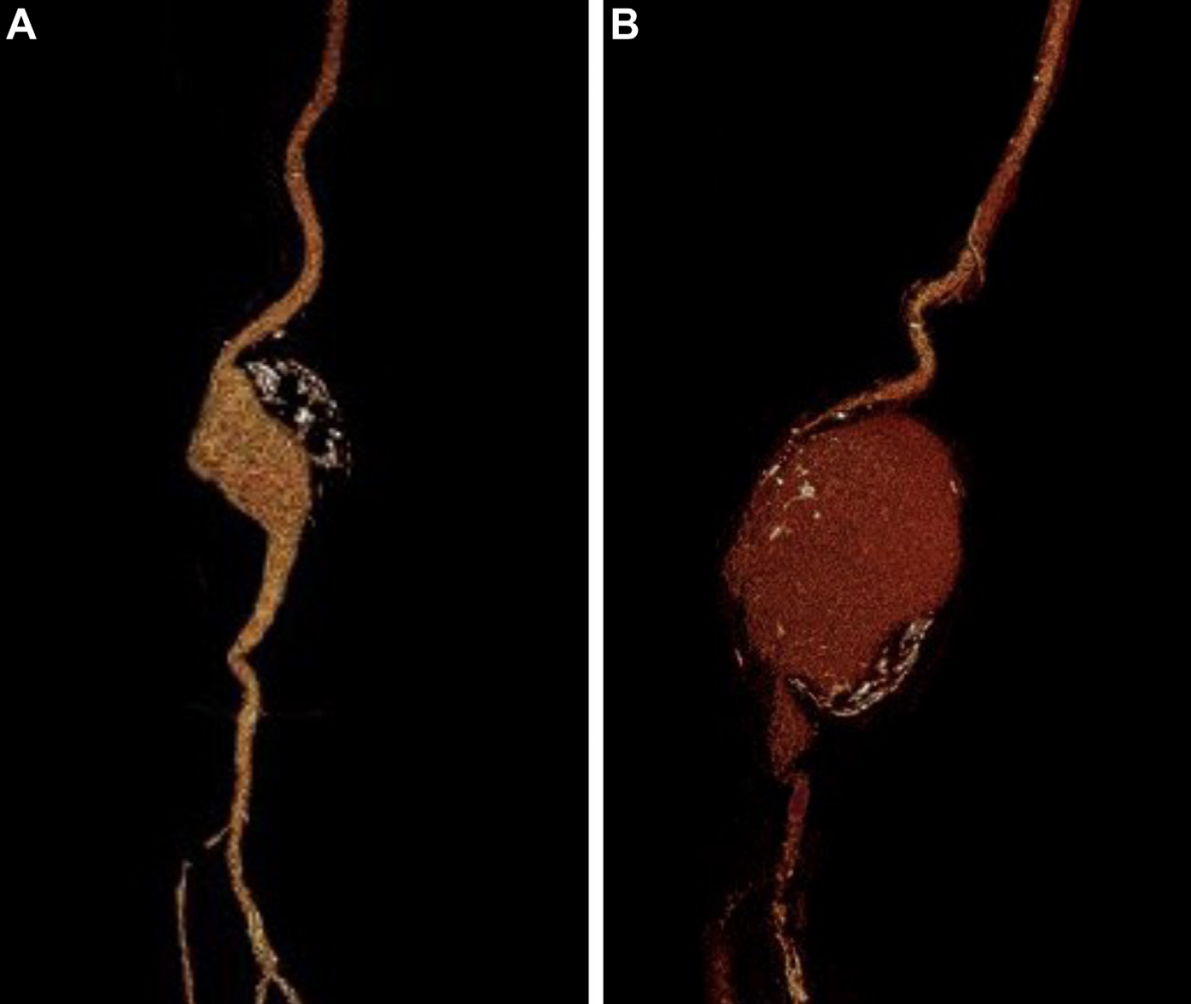
Three-dimensional reconstructions of the right popliteal artery aneurysm (PAA) demonstrating rapid expansion from 46 mm **(A)** to 81 mm **(B)** within a 2-week period. The aneurysm diameter was measured on axial images perpendicular to the flow line to the largest diameter of the vessel with three-dimensional reconstructed computed tomography images.

A previously healthy 63-year-old white man who worked daily with horses was admitted to our hospital with a 2-week history of progressive right leg swelling and pain associated with intermittent low-grade fever. Two weeks earlier, he had had an episode of septic arthritis from his right knee with growth of *Streptococcus equi* on cultures of synovial fluid and blood. This was sensitive to all tested antibiotics, including amoxicillin, cefadroxil, cefotaxime, erythromycin, isoxazolyl penicillin, clindamycin, benzylpenicillin, and penicillin V. He had no history of trauma, and the two blood cultures taken at the current admission again revealed growth of *Streptococcus equi*. The screening tests for human immunodeficiency virus and hepatitis were negative. Because of the progressive swelling of the right leg, duplex ultrasound (DUS) was performed, showing a deep vein thrombosis, which led to initiation of direct oral anticoagulant therapy with dabigatran etexilate. Orthopedic consultation waived the need for a new diagnostic puncture owing to the quick resolution of the swelling after initiation of direct oral anticoagulant therapy. No vegetations were seen on a transthoracic echocardiogram. Computed tomography angiography was uneventful, except for a partially thrombosed fusiform popliteal artery aneurysm (PAA) on the right leg, extending from P1 to P2, with a diameter of 46 × 55 mm and three open runoff vessels below the knee (Figs 1 and 2). After 14 days of intravenous benzylpenicillin, the patient was discharged from hospital, given the improvement in his symptoms, with a plan for 6 weeks of oral amoxicillin and an expedited follow-up at the outpatient clinic to plan for PAA repair. He was readmitted 3 days later because of recurrence of intermittent fever and progressive right leg swelling and pain. He was tachycardic and normotensive, with a temperature of 38.1°C. Pitting edema of the right leg was present. The right leg was also tender, without any overlying skin changes. No signs of ischemia or sensory or motor loss or deficit were present.

**Fig 3:**
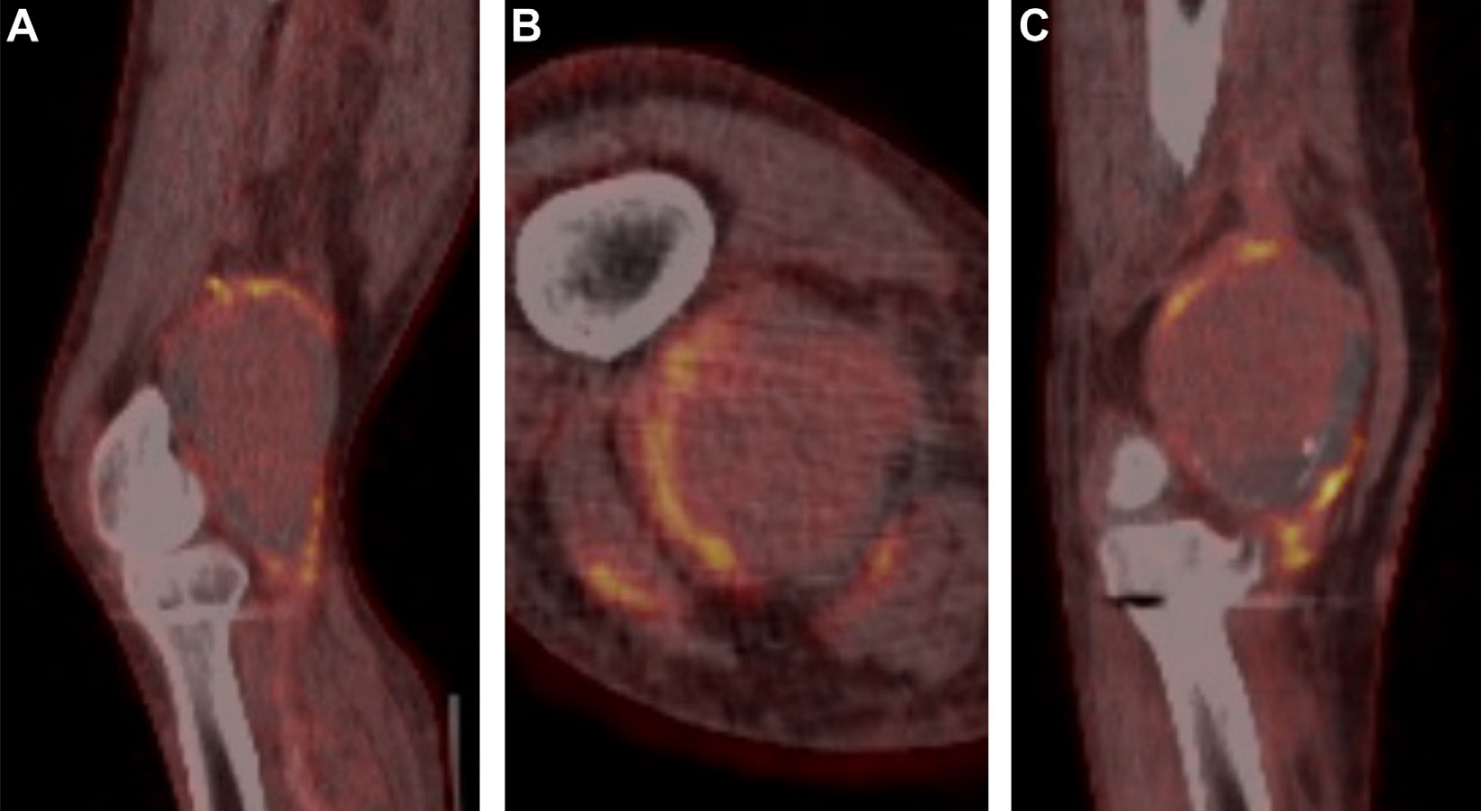
Fluorine-18 fluorodeoxyglucose positron emission tomography/computed tomography images showing hypermetabolic activity on the arterial wall of the large infective popliteal artery aneurysm (PAA) on coronal **(A)**, axial **(B)**, and sagittal **(C)** projections.

The laboratory blood tests showed an elevated white blood cell count (8.9 × 10^9^/L), C-reactive protein (139 mg/L), and erythrocyte sedimentation rate (112 mm/h). New blood cultures were negative. A new computed tomography angiogram of the lower limbs revealed an expansion of the PAA to a maximal diameter of 81 × 66 mm with patent runoff vessels on the lower limb. The suspicion of an infective PAA was reinforced by the hypermetabolic activity around the large PAA on fluorine-18 fluorodeoxyglucose positron emission tomography/computed tomography (Fig 3).

A femoropopliteal bypass with a reversed great saphenous vein from the left leg via a medial approach was performed, followed by almost complete resection of the aneurysm sac from the same access site. To mobilize the aneurysm and freely dissect to a healthy vessel segment, complete release of the medial gastrocnemius muscle from the femoral condyle was performed, as well as release of the tendons in the pes anserine. These were all reinserted with tendon sutures at the finalization of the vascular reconstruction.

The intraoperative cultures and 16s rDNA bacterial DNA sequencing analysis obtained from the aneurysm sac were consistent with *Streptococcus equi*. The postoperative course was uneventful, with the exception of a sensory motor deficit of the right leg, with neurography confirming sensorimotor polyneuropathy with a deficit on the peroneus nerve. This was considered iatrogenic and most likely due to mobilization of the medial gastrocnemius muscle and the extent of the phlegmon in the dissection site. DUS after 30 days showed a patent bypass with no signs of stenosis. The patient was discharged on postoperative day 31 with partial recovery of the neurologic deficit and a 3-month prescription for oral clindamycin.

At the 1-month follow-up visit after discharge, the patient had almost complete recovery from the peroneus nerve damage after continued rehabilitation and ≥5000 steps/d as daily activity. A new DUS confirmed patency without stenosis of the revascularized limb. An intensive follow-up schedule with clinical and laboratory assessments was planned, with a minimum of 6 months of antibiotic therapy.

## Discussion

*Streptococcus equi*, subspecies *Zooepidemicus*, is a species of gram-positive, coccoid bacteria isolated from abscesses in submaxillary glands and mucopurulent discharge of the upper respiratory tract of horses. It is an opportunistic pathogen for both humans and a broad range of species, including horses, dogs, and pigs.^4^

To the best of our knowledge, the present case is the first case reported of a symptomatic infective PAA with a pathogen most commonly known to affect equids such as *Streptococcus equi* as the infectious pathogen. The patient’s anamnesis and background revealed his potential exposure to this unusual pathogen for human infections because he worked daily with horses. Moreover, the recent anamnesis of septic arthritis with the same pathogen should raise awareness for this rare colonization of the PAA. The directness of this course of the disease could not be ascertained completely because the patient had most likely been contaminated with the pathogen through the respiratory track via inhalation while working near horses. This could have led to bacteremia and subsequently seeded the knee joint and the PAA.

The deep vein thrombosis was certainly a contributor to the patient's leg swelling. Its cause was most likely multifactorial, with compression of the popliteal vein by the rapidly expanding infective PAA. In addition, the local inflammation and bacteremia could have played a role. Venous DUS is the most common imaging modality of choice in the diagnostic evaluation of a patient with unilateral lower swelling and pain. However, in cases of potential infectious seeding, fluorine-18 fluorodeoxyglucose positron emission tomography/computed tomography can be very useful, as it was for the present case. The uptake was not limited to a perivenous area but instead to the aneurysm sac. Moreover, 16s rDNA sequencing analysis of bacterial taxonomy can be particularly useful, especially for patients already receiving antibiotic therapy. In retrospect, a more expedited diagnostic workup after the initial intravenous antibiotic course would have allowed for an earlier diagnosis and repair of the infective PAA without the final expansion.

Open surgical repair with autologous material and debridement remains the treatment of choice for infective PAAs when the anatomy is favorable. Endovascular treatments have been described and still have a role but mainly for high-risk patients.^5^^,^^6^ Because no specific treatment guidelines are available, management is commonly guided by the general principles of vascular surgery. No consensus has been reached in the literature regarding the approach for open revascularization, with medial and posterior approaches used equally and each offering separate advantages and disadvantages. The decision should be individualized; however, with local infection, there is increased difficulty with the posterior approach owing to the increased risk of bleeding and nerve damage. For our patient, the first issue was controlled by excluding the aneurysm before resection; however, temporary nerve damage still occurred.

## Conclusions

An infective PAA is a rare, but potentially devastating, condition. A high index of suspicion and expedited diagnostic workup is, therefore, necessary, especially in the case of rapid enlargement of a PAA with an anamnesis of recent infections. A careful anamnesis taking could reinforce the suspicion of zoonosis even if not previously described, as in the present case. Surgical reconstruction using an autologous vein graft seems to be a feasible solution. Prolonged targeted antibiotic therapy and close follow-up protocols are recommended.
